# Functional Specificity of Sex Pheromone Receptors in the Cotton Bollworm *Helicoverpa armigera*

**DOI:** 10.1371/journal.pone.0062094

**Published:** 2013-04-15

**Authors:** Yang Liu, Chengcheng Liu, Kejian Lin, Guirong Wang

**Affiliations:** State Key Laboratory for Biology of Plant Diseases and Insect Pests, Institute of Plant Protection, Chinese Academy of Agricultural Sciences, Beijing, China; Plant and Food Research, New Zealand

## Abstract

Male moths can accurately perceive the sex pheromone emitted from conspecific females by their highly accurate and specific olfactory sensory system. Pheromone receptors are of special importance in moth pheromone reception because of their central role in chemosensory signal transduction processes that occur in olfactory receptor neurons in the male antennae. There are a number of pheromone receptor genes have been cloned, however, only a few have been functionally characterized. Here we cloned six full-length pheromone receptor genes from *Helicoverpa armigera* male antennae. Real-time PCR showing all genes exhibited male-biased expression in adult antennae. Functional analyses of the six pheromone receptor genes were then conducted in the heterologous expression system of *Xenopus* oocytes. HarmOR13 was found to be a specific receptor for the major sex pheromone component Z11-16:Ald. HarmOR6 was equally tuned to both of Z9-16: Ald and Z9-14: Ald. HarmOR16 was sensitively tuned to Z11-16: OH. HarmOR11, HarmOR14 and HarmOR15 failed to respond to the tested candidate pheromone compounds. Our experiments elucidated the functions of some pheromone receptor genes of *H. armigera*. These advances may provide remarkable evidence for intraspecific mating choice and speciation extension in moths at molecular level.

## Introduction

Male moth can accurately find and recognize mates through detection of the sex pheromone at extremely low concentration emitted from conspecific females. Notably, male moths can discriminate a subtle difference in stereochemistry or chirality of molecules or ratio change of two or a few components as a species-specific cue [1], [2], [3]. The outstanding sensitivity and specificity of moth pheromone detection has made the male moth antenna an attractive model system in animal olfactory research [4], [5], . Since the first sex pheromone was discovered from the silkmoth, *Bombyx mori* [2], sex-pheromone components in more than 500 insect species, including many important agricultural pests, have been identified [8]. With the development of DNA sequencing technology, more and more sex pheromone receptor genes have been cloned, however, only a few have been functionally characterized.

Intensive studies have been focused on the silkworm moth *B. mori* since it has the simplest pheromone components and its whole genome sequence was released in 2004 [1], [9], [10], [11], [12], [13]. The pheromone sensitive trichiod sensilla are in the majority amongst several different types of sensilla on male silkworm antennae [14], [15]. Each trichiod sensilla houses two pheromone-sensitive neurons: one of which coexpresses BmOR1/BmOR2 that is activated by bombykol, the major pheromone that elicits the male mating behavior [16]. The other neuron coexpresses BmOR3/BmOR2, which binds to bombykal, which has an inhibitory effect on male behavior response [1]. In addition to *B.mori*, much research on pheromone reception has been performed in the genus *Ostrinia*. Most species, including European corn borers (ECB), in the genus *Ostrinia* use varying ratios of Z11- and E11-tetradecenyl acetate (Z11- and E11-14:OAc) as the two main components of their pheromone blend. At least five different sex pheromone receptor candidates have recently been identified and functionally characterized in vitro using *Xenopus* oocytes system. Except that ECB(Z) OR6 responded almost exclusively to Z11, the primary pheromone produced by ECB(Z) females, other receptors responded broadly to sex pheromone components in general [17], [18], [19].

Additionally, the genus *Heliothis* and closely related species including *Helicoverpa armigera* and *Helicoverpa assulta* are also good model systems for studying the evolution of the pheromone biosynthesis and perception systems since sex pheromone components of most species were identified and various species show distinct differentiation in sex pheromones components or ratios. All six sex pheromone receptor candidates in *Heliothis virescens* were identified by using a combination of genomic sequence analysis, cDNA-library screening as well as BAC library sequence and further functional characterization in heterologous expression systems, such as *Xenopus* oocytes or HEK293 cell culture [6], [20], [21]. The receptors' functional activity is closely associated with pheromone-sensitive neuronal function from single male sensillum electrophysiological recordings [22], [23]. HvOR13 in A-type sensillum and HvOR6 in B-type sensillum specifically are tuned to Z11-16:Ald, the major pheromone component and Z9-14:Ald, the second pheromone component, respectively. HvOR14 and HvOR16 in C-type sensillum are, respectively, tuned to Z11-16:OAc and Z11-16:OH [21]. At this point, the function of pheromone receptors from the genus *Heliothis* and closely related species are still largely unknown.

The cotton bollworm *H.armigera* (Hübner) (Lepidoptera: Noctuidae) is a notorious agricultural pest worldwide [24]. One of the most important sex pheromone component for the species is (Z)-11-hexadecenal (Z11-16:Ald), which alone can attract a small number of male moths. Z9-16:Ald is generally thought to be an important minor component [25], [26], [27], [28], [29]. Adding Z9-16:Ald to Z11-16:Ald with ratio from 1∶99 to 10∶90 caused a significant increase in trap catch of male *H. armigera* [27], [30], [31]. Many other components were also identified in sex pheromone glands of female *H. armigera*, including tetradecanal (14:Ald), (Z)-11-tetradecenol (Z11-14:OH), (Z)-9-tetradecanal (Z9-14:Ald), 16:Ald, Z7-16:Ald, 16:OH, Z9-16:OH, Z11-16:OH, Z11-14:Ald and Z11-16:Ac [27], [29], [32], [33]. Of these, Z11-16:OH and 16:OH are attraction inhibitors when added to attraction blends [27], [29], [30]. Z9-14:Ald was shown to increase attraction in low concentration and inhibit in high concentration in combination with other compounds [27], [32], [34], [35].

Electrophysiological recordings from single male sensillum have demonstrated that there are at least two types of pheromone-responsive sensilla in male antennae tuned to Z11-16:Ald and Z9-16:Ald, respectively [36]. At a molecular level, six Heliothis-homologous pheromone receptor candidates were identified using transcriptomic analysis [37], [38]. However, we know nothing about their functions.

In this study, we cloned six full-length pheromone receptor genes from *H. armigera* male antennae, which appeared to be orthologs of *H. virescens* pheromone receptor genes, respectively. Expression patterns of these pheromone receptor genes were evaluated by quantitative real-time PCR showing all genes exhibited male-biased expression in adult antennae. Finally, we characterized the functional properties of some of these pheromone receptor genes in the heterologous expression system of *Xenopus* oocytes.

## Methods

### Insect

The *H. armigera* colony was maintained at the Institute of Plant Protection, Chinese Academy of Agricultural Sciences, Beijing, China. Larvae were reared on an artificial diet at 27±1°C with a photoperiod of 14∶10 (L:D). Pupae were selected by sex and placed in separate test tubes. Different tissues of male and female adults were excised at the base at 1–3 days after eclosion and immediately frozen in liquid nitrogen, and then stored at under −70°C until use.

### Pheromone components

(Z)-11-hexadecenal (Z11-16:Ald) and (Z)-11-hexadecen-1-ol (Z11-16:OH) (both 95% minimum purity) were purchased from Nimrod Inc. (Changzhou, China). (Z)-9-tetradecenal (Z9-14:Ald), (Z)-9-hexadecenal (Z9-16:Ald), (Z)-7-hexadecenal (Z7-16:Ald), (Z)-11-hexadecenyl acetate (Z11-16:OAC) and hexadecenal (16:Ald) (all 93–95% minimum purity) were purchased from Bedoukian (Danbury, CT, USA). Stock solutions (1 M) were prepared in dimethyl sulfoxide (DMSO) and stored at −20°C. Before experiments, the stock solution was diluted in 1× Ringer's buffer (96 mM NaCl, 2 mM KCl, 5 mM MgCl2, 0.8 mM CaCl2 and 5 mM HEPES pH 7.6). 1× Ringer's buffer containing 0.1% DMSO was used as a negative control.

### RNA extraction and cDNA synthesis

The frozen tissue was transferred to a liquid nitrogen-cooled mortar and ground. The homogenate was covered with 1 mL of TriZol reagent (Invitrogen, Carlsbad, CA, USA). Further steps were performed according to the manufacturer's instruction. Total RNA was dissolved in RNA-free ddH_2_O. RNA quantity and integrity were determined on a Nanodrop ND-1000 spectrophotometer (Nano-Drop products, Wilmington, DE, USA) and gel electrophoresis. Prior to cDNA synthesis, RNA was treated with DNase I (Fermentas, Vilnius, Lithuania) to remove trace amounts of genomic DNA. The cDNA was synthesized by First Strand cDNA Synthesis Kit (Fermentas, Vilnius, Lithuania) and was used as a template in PCR reactions.

### Cloning of full-length cDNA encoding pheromone receptors in *H.armigera*

The sequences of six pheromone receptor genes and an Orco gene were identified through antennal transcriptomic analysis [38]. Four sequences including *HarmOR11, HarmOR13, HarmOR16 and HarmOR2* were verified as full-length open-reading frames (ORFs) using RACE amplification in a previous report [37]. The full-length ORFs of the other three genes were verified in this study using SMARTer™ RACE cDNA Amplification Kit (Clontech, Mountain View, CA, USA) following the user manual. Finally, full-length coding sequences of candidate pheromone receptor genes and Orco gene of *H.armigera* were PCR amplified from pools of total cDNA prepared from male antennae using primeSTAR HS DNA polymerase (Takara, Dalian, China). PCR reactions of 25 µl contained 0.25 µl primeSTAR HS DNA polymerase, 5 µl 5×PrimerSTAR Buffer (Mg^2+^ plus), 2 µl dNTP mixture (2.5 mM) and 0.5 µl of each primer (10 µM). All amplification reactions were carried out using a Veriti Thermal Cycler (Applied Biosystems, Carlsbad, CA, USA) under the following conditions: 94°C for 2 min; 34 cycles of 94°C for 30 s, 55–60°C for 30 s, 72°C for 1.5 min; and 72°C for 10 min. PCR amplification products were run on a 1.0% agarose gel and then verified by DNA sequencing. The primer sequences used in this study were listed in Material S1.

### Sequence analysis

Multiple alignments and identity calculation were done with the ClustalW2 [39] software. The phylogenetic reconstruction implemented for the analysis of HarmPRs was performed based on the amino sequences of the six *H. armigera* PRs and the OR sequences identified in Lepidoptera (21 from *H. virescens* and 64 from *B. mori*) [20], [40], [41]. Unrooted trees were constructed by the neighbor-joining with p-distance method, as implemented in MEGA5 software [42]. For transmembrane domain predictions the TMHMM Server Version2.0 (http://www.cbs.dtu.dk/services/TMHMM) was used.

### Quantitative real-time PCR

Total RNAs were extracted from antennae(A), heads without antennae (H), thoraxes (T), maxillary palps (MP), proboscises (PR), abdomens (AB), legs (L) and genitals (G). The qRT-PCR template were synthesized as described above and the negative reactions (all reagents without reverse polymerase) were sited to make sure there are no gDNA residual.. Primers were designed using the Primer Premier 5 software (PREMIER Biosoft International). The primer sequences are listed in Material S1. The annealing temperatures of the primers were controlled at 62±3°C. The efficiency of primer was calculated by standard curve method. The efficiency of all the primers was ranging from 0.9–1.1. The qRT-PCR was performed following the manufacturer's instruction of the SYBR II Premix Ex Taq kit (Takara, Dalian, China) with a MyiQ2 Two-Color Real-Time PCR Detection System (Bio-Rad, Hercules, CA, USA). The PCR procedure was as followed: one cycle of 95°C for 30 s; 40 cycles of 95°C for 10 s, 55°C for 30 s (read); 95°C for 1 min, 55°C for 1 min. A dissociation curve was used to ensure primer specificity and lack of contamination. qRT-PCR products were analysed by 2% agarose gel electrophoresis and then verified by DNA sequencing. The housekeeping gene- actin was used as control. The experiment was repeated three times using three independent RNA samples. The expression level of the pheromone receptor genes was analyzed using th 2^−ΔΔCT^ method. where ΔCT = (CT, PR gene–CT, actin gene), ΔΔCT = (ΔCT, different samples −ΔCT maximum) [43]. Statistical comparison of expressions of the pheromone receptor genes was assessed using a single classification ANOVA procedure. The data were presented as mean±SE and the images were optimized by GraphPad Prism 5.

### Receptor expression in *Xenopus* oocytes and electrophysiological recordings

Receptor expression and electrophysiological recording were performed as decribed in previous reports [44], [45]. The full-length cDNA sequences of pheromone receptors were first cloned into pENTR/D-TOPO entry vectors (Invitrogen, Carlsbad, CA, USA) and then subcloned into pSP64T (converted from pSP64T-Oligo) destination vectors by means of the Gateway LR reaction. cRNAs were synthesized from linearized vectors with mMESSAGE mMACHINE SP6 (Ambion, Austin, TX, USA). Mature healthy *Xenopus* oocytes (stage V–VII) were separated and then treated with 2 mg/ml collagenase I in washing buffer (96 mM NaCl, 2 mM KCl, 5 Mm MgCl2, and 5 mM HEPES, pH 7.6) for 1–2 h at room temperature. The mixture of 27.6 ng pheromone receptor cRNA each and 27.6 ng HarmOrco (HarmOR2) cRNA Oocytes were later microinjected into the oocytes. The *Xenopus* oocytes were incubated for 4–7 days at 18°C in incubation medium (1× Ringer's buffer, 5% dialysed horse serum, 50 mg/ml tetracycline, 100 mg/ml streptomycin and 550 mg/ml sodium pyruvate) after injection. Whole-cell currents of the injected oocytes were recorded with a two-electrode voltage clamp. Odorant induced currents were recorded with an OC-725C oocyte clamp (Warner Instruments, Hamden, CT, USA) at a holding potential of −80 mV. The data were acquired and analyzed with Digidata 1440A and Pclamp10.0 software (Axon Instruments Inc., Union City, CA, USA). Dose–response data were analysed using GraphPad Prism 5.

## Results

### cDNA cloning and sequence analysis of pheromone receptor genes in the *H. armigera*

**Figure 1:**
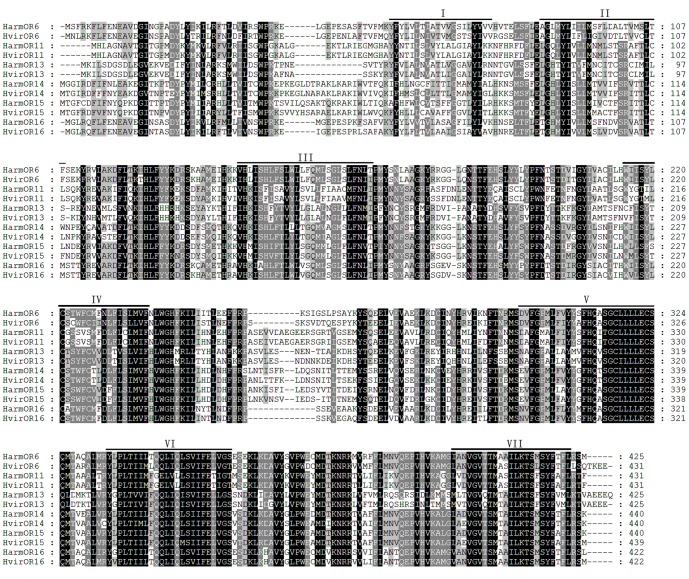
Amino acid sequence alignments of the *H. armigera* and *H. virescens* PRs. Predicted seven-transmembrance domains are identified with roman numbers. Amino acid numbering is given on the right of the alignment. Gaps in the alignment are indicated by a dash.

**Figure 2:**
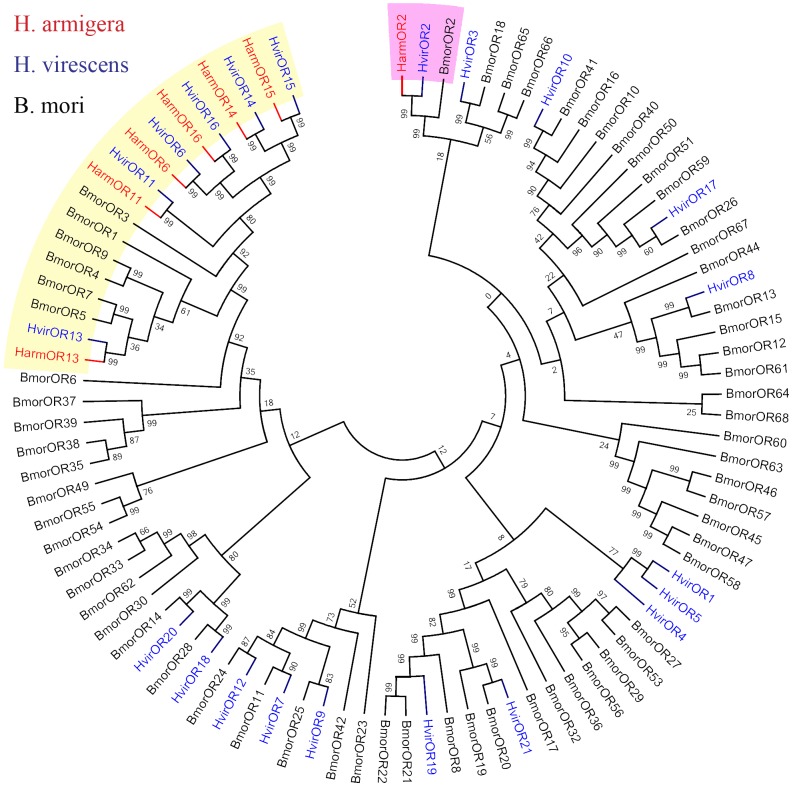
Phylogenetic tree of the *H. armigera* PRs and other lepidopterans ORs. Harm: *H. armigera* (red), Hvir: *H. virescens* (blue), Bmor: *B. mori* (black). The clade of PRs was masked by yellow shadow. The clade of Orco was masked by pink shadow.

Six cDNA sequences encoding *H. armigera* pheromone receptor genes were identified (GenBank accession number: KC538876-KC538881) and three of these sequences including *HarmOR11, HarmOR13 and HarmOR16* were verified as full-length open-reading frames in previous reports [37], [38]. The full-length ORFs of other three genes including *HarmOR6*, *HarmOR14 and HarmOR15* were obtained using RT-PCR and 5′/3′-RACE amplification. The lengths of amino acid residues encoded by these pheromone receptor genes ranged from 425 to 440 amino acid residues and were predicted to possess seven transmembrane domains. Multiple alignments of *H. armigera* PRs and their homologs in *H. virescens* showed the orthologous PRs in these two insects had high similarity. The identities between the two species of OR6, OR11, OR13, OR14, OR15, and OR16 were 80.3%, 95.8%, 90.9%, 84.1%, 83.6% and 88.2%, respectively (Figure 1). The phylogenetic analyses showed that these PRs were clustered together with other lepidopteran PRs separated from general odor receptors (Figure 2).

### Tissue-specific and male-biased expression of six sex pheromone receptor genes

**Figure 3:**
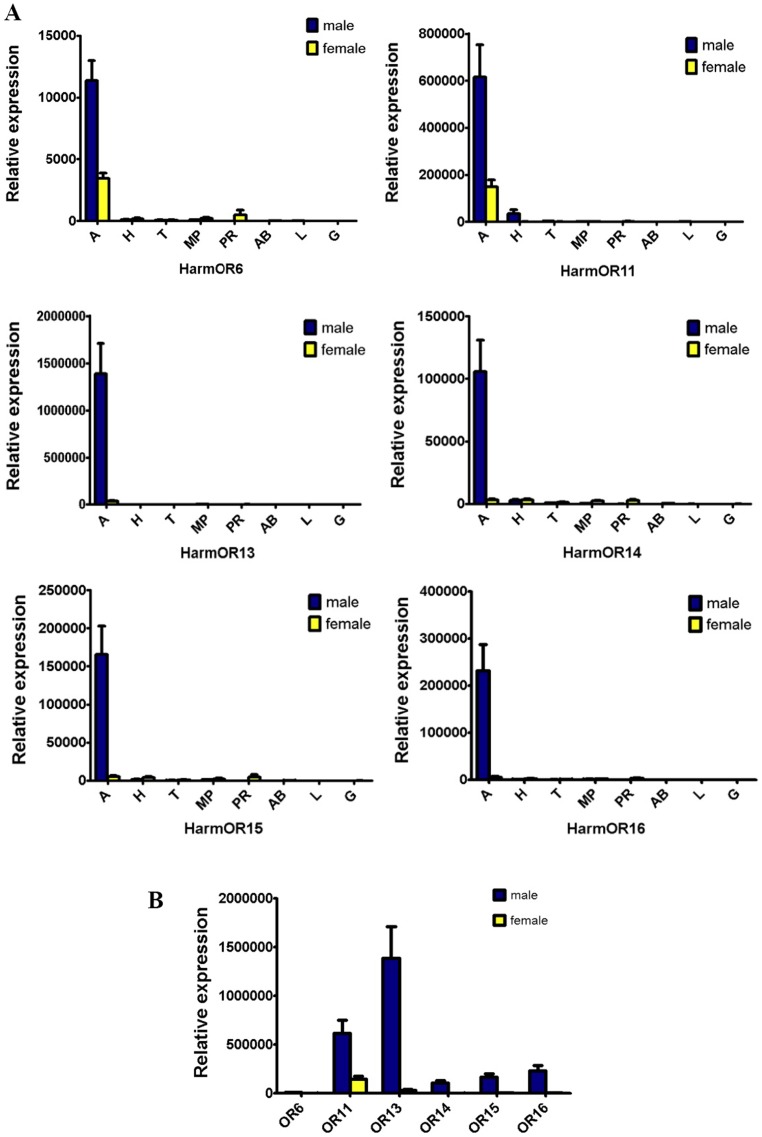
Tissue- and sex-specific expression of the *H. armigera* PRs. A: Expression of the *H. armigera* PRs in eight tissues of two sexes including antennae (A), heads (H), thoraxes (T), maxillary palps (MP), proboscises (PR), abdomens (AB), legs (L) and genitals (G). B: Comparison of PR expression between male and female antenna of *H.armigera*. Error bars indicate SE.

The average values of expression levels of all sex pheromone receptor genes are from three biological replications by quantitative real-time PCR. All sex pheromone receptor genes were expressed in male antennae with higher levels compared to female antennae and the ratio (male: female) of HarmOR6, HarmOR11, HarmOR13, HarmOR14, HarmOR15 and HarmOR16 was 3.35±0.85, 4.17±0.66, 41.43±2.79, 35.99±8.85, 32.22±6.61 and 42.05±2.56 (Figure 3A). In male antennae, HarmOR13 was detected as the most abundant, 2.24±0.15, 5.98±0.62, 8.30±0.27 and 13.15±1.86 times higher than HarmOR11, 16, 15 and 14, respectively. HarmOR6 was detected at the lowest level and was approximately two orders of magnitude less abundant than HarmOR13 (Figure 3B). Generally speaking, all sex pheromone receptor genes were very weakly expressed, if at all, in other tissues such as heads, legs, abdomen and mouthparts except for HarmOR11 showing significant expression at male, but not female heads.

### HarmOR13 is a specific receptor for the major sex pheromone component

**Figure 4:**
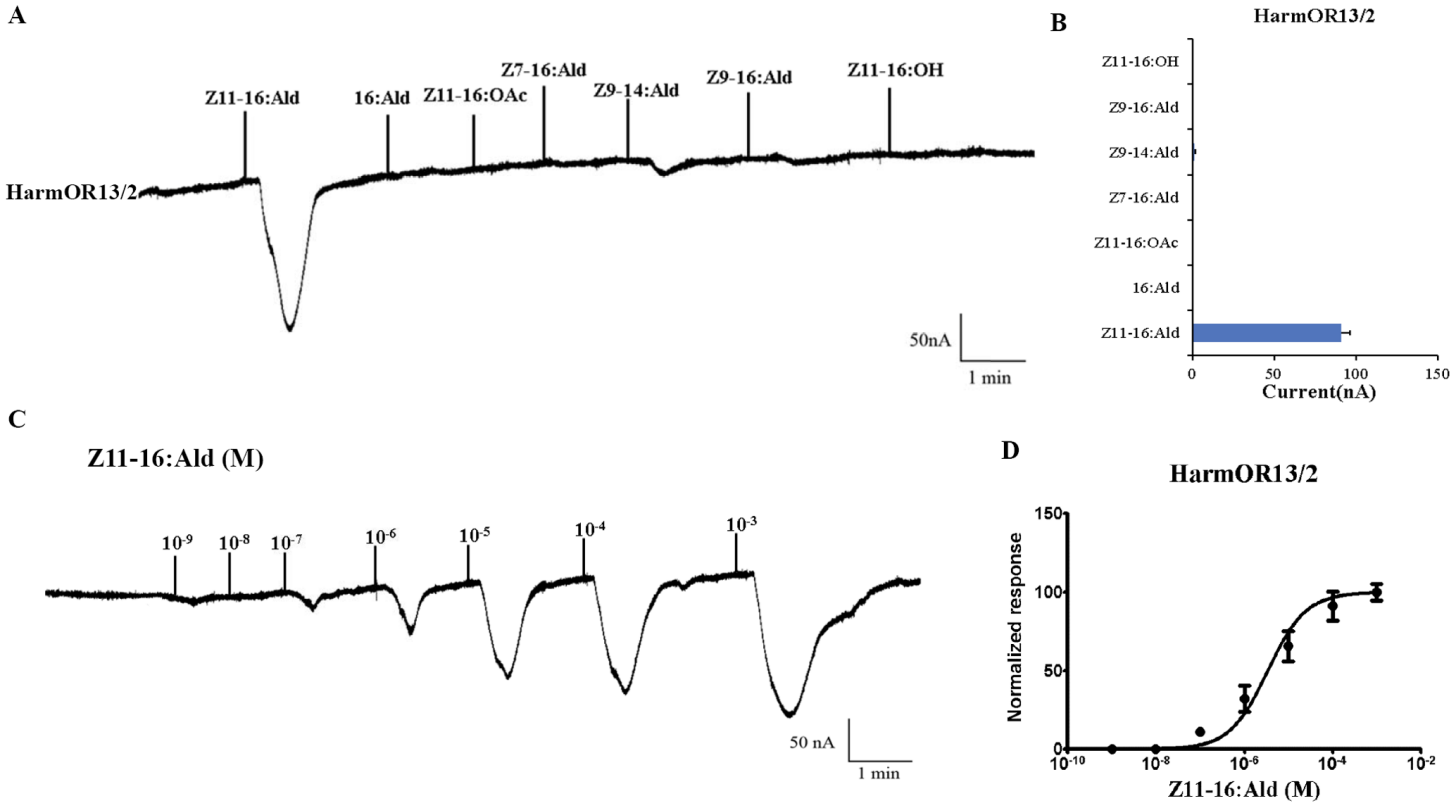
Responses of *Xenopus* oocytes with co-expressed HarmOR13/HarmOR2 to stimulation with pheromone compounds. (A) Inward current responses of HarmOR13/HarmOR2 *Xenopus* oocytes in response to 10^−4^ M solution of pheromone compounds. (B) Response profile of HarmOR13/HarmOR2 *Xenopus* oocytes. Error bars indicate SEM (n = 7). (C) HarmOR13/HarmOR2 *Xenopus* oocytes stimulated with a range of Z11-16:Ald concentrations. (D) Dose–response curve of HarmOR13/HarmOR2 *Xenopus* oocytes to Z11-16:Ald. Responses are normalized by defining the maximal response as 100. EC50 = 3.403×10^−6^ M. Error bars indicate SEM (n = 6).

Each of the six pheromone receptors were co-expressed in *Xenopus* oocytes with the obligatory functional chaperone HarmOrco for 5–7 days [1], [21] followed by testing using a panel of candidate *H. armigera* pheromone compounds including the major and second sex pheromone components (Z11-16:Ald and Z9-16:Ald). The oocytes co-expressing HarmOR13 and HarmOrco robustly responded to 10^−4^ M Z11-16: Ald and yielded little if any response to Z9-14:Ald. In dose-response studies, 10^−7^ M Z11-16:Ald could elicit significant responses from oocytes that co-expressed HarmOR13 and HarmOrco and the EC50 value was 1.82×10^−6^ M. No responses were elicited in similar tests or with even higher concentrations (10^−3^ M) of Z9-16:Ald, 16:Ald, Z11-16:OAc, Z7-16:Ald, Z11-16:OH. (Figure 4)

### HarmOR6 is equally tuned to both of Z9-16: Ald and Z9-14: Ald

**Figure 5:**
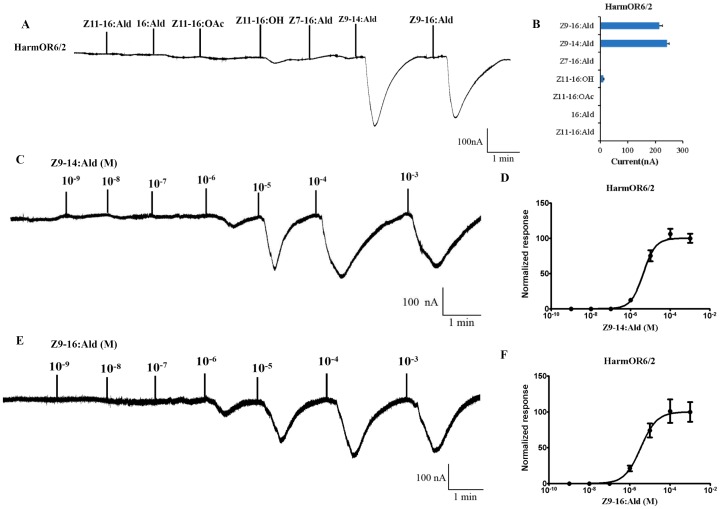
Responses of *Xenopus* oocytes with co-expressed HarmOR6/HarmOR2 to stimulation with pheromone compounds. (A) Inward current responses of HarmOR6/HarmOR2 *Xenopus* oocytes in response to 10^−4^ M solution of pheromone compounds. (B) Response profile of HarmOR6/HarmOR2 *Xenopus* oocytes. Error bars indicate SEM (n = 7). (C) HarmOR6/HarmOR2 *Xenopus* oocytes stimulated with a range of Z9-14:Ald concentrations. (D) Dose–response curve of HarmOR6/HarmOR2 *Xenopus* oocytes to Z9-14:Ald. Responses are normalized by defining the maximal response as 100. EC50 = 4.338×10^−6^ M. Error bars indicate SEM (n = 6). (E) HarmOR6/HarmOR2 *Xenopus* oocytes stimulated with a range of Z9-16:Ald concentrations. (F) Dose–response curve of HarmOR6/HarmOR2 *Xenopus* oocytes to Z9-16:Ald. Responses are normalized by defining the maximal response as 100. EC50 = 3.531×10^−6^ M. Error bars indicate SEM (n = 6).

*Xenopus* oocytes co-expressing HarmOR6 and HarmOrco responded robustly to both Z9-16:Ald and Z9-14:Ald and yielded little if any response to Z11-16:OH (Figure 5A,B). We next conducted dose-response analysis to elucidate if *Xenopus* oocytes co-expressing HarmOR6 and HarmOrco have significantly different sensitivity to these two ligands. Surprisingly, *Xenopus* oocytes co-expressing HarmOR6 and HarmOrco not only equally responded to 10^−4^ M of Z9-16:Ald and Z9-14:Ald, but also possessed similar sensitivity to these two ligands and the EC50 to Z9-16:Ald and Z9-14:Ald were 3.53×10^−6^ M and 4.34×10^−6^ M, respectively (Figure 5C–F).

### HarmOR16 is sensitively tuned to Z11-16: OH

**Figure 6:**
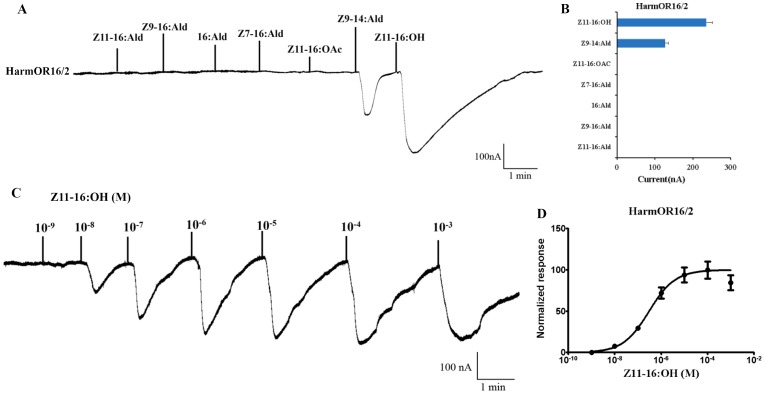
Responses of *Xenopus* oocytes with co-expressed HarmOR16/HarmOR2 to stimulation with pheromone compounds. (A) Inward current responses of HarmOR16/HarmOR2 *Xenopus* oocytes in response to 10^−4^ M solution of pheromone compounds. (B) Response profile of HarmOR16/HarmOR2 *Xenopus* oocytes. Error bars indicate SEM (n = 7). (C) HarmOR16/HarmOR2 *Xenopus* oocytes stimulated with a range of Z11-16:OH concentrations. (D) Dose–response curve of HarmOR16/HarmOR2 *Xenopus* oocytes to Z11-16:OH. Responses are normalized by defining the maximal response as 100. EC50 = 2.988×10^−7^ M. Error bars indicate SEM (n = 6).

**Figure 7:**
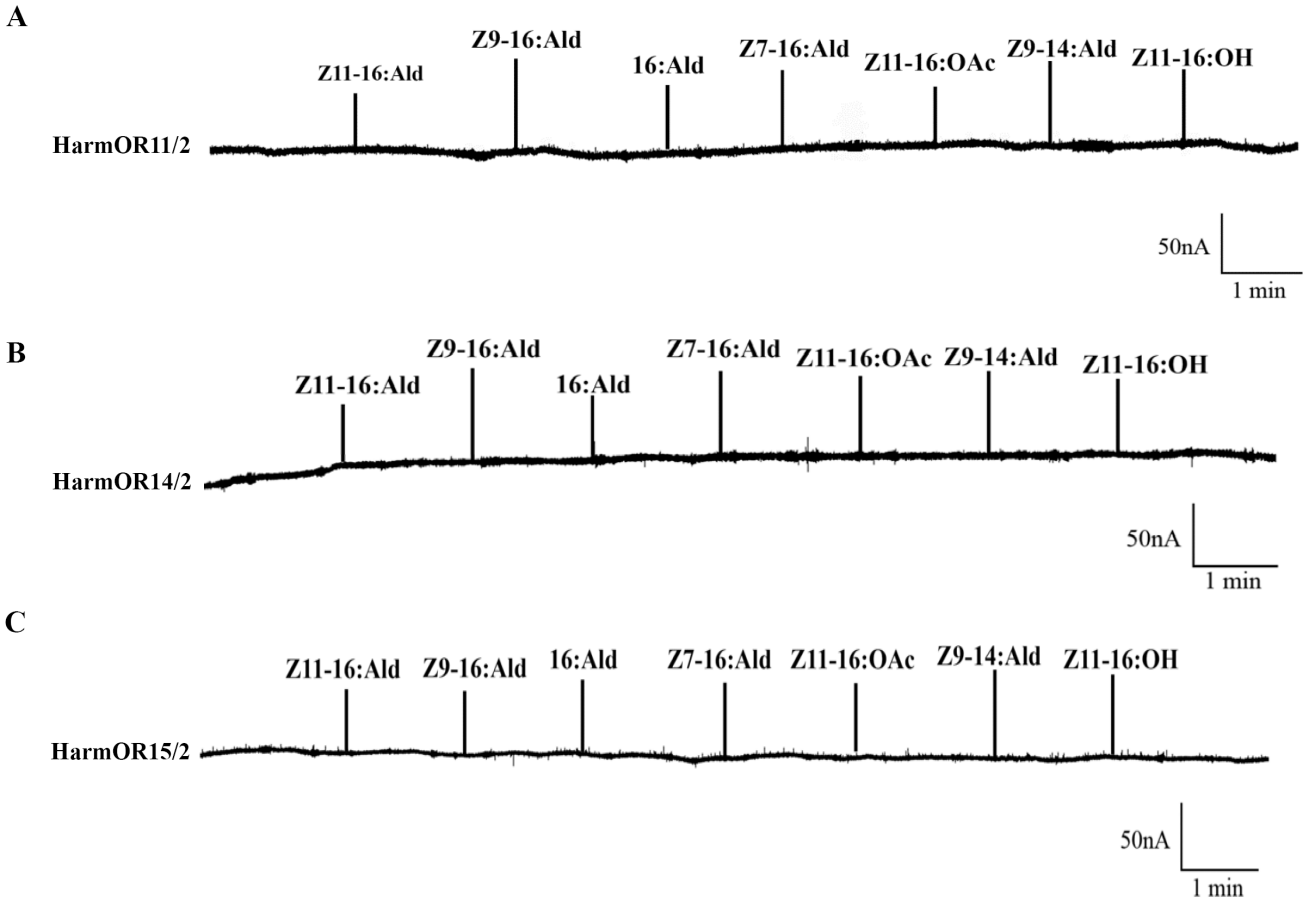
No ligand was identified of three candidate pheromone receptor genes in *H.armigera*. (A) HarmOR11/HarmOR2. (B) HarmOR14/HarmOR2 and (C) HarmOR15/HarmOR2. The concentrations of all tested pheromone compounds were 10^−4^ M.

*Xenopus* oocytes co-expressing HarmOR16 and HarmOrco strongly responded to Z11-16:OH and have a mean amplitude response of 233nA. In the dose response studies, oocytes had measurable responses as low as 10^−8^ M and the EC_50_ to Z11-16:OH was 3.0×10^−7^ M. *Xenopus* oocytes co-expressing HarmOR16 and HarmOrco also had significant response to 10^−4^ M Z9-14:Ald (Figure 6). The other three candidate pheromone receptors including HarmOR11, HarmOR14 and HarmOR15 failed to respond to the tested candidate pheromone compounds (Figure 7).

## Discussion

Sex pheromone communication between male and female moths is believed to have contributed to successful intraspecific mating choice and interspecific isolation [46]. The behavioral response of male moths to sex pheromone has been proven to be closely linked to the activity of the peripheral olfactory receptor neurons (ORNs) [1], [17]. The peripheral ORNs of male moths, which are tuned to sex pheromone components, are housed within long sensilla trichodea in the antennae usually in groups of two or three ORNs in each sensillum. Recent studies revealed that in many, if not all, cases the odor receptor was the primary determinant of the odor response spectrum of the peripheral ORN [21], [47], [48]. The cotton bollworm, *H. armigera* and its closely related species, such as *H. assulta and Heliothis* spp. have similar sex pheromones components with tiny differentiations in minor components or ratios. Therefore, elucidating the functions of pheromone receptor genes will provide remarkable evidence for intraspecific mating choice and speciation extension in moths at molecular level.

In this study, we identified and cloned the full-length ORFs of six sex pheromone receptor genes from *H. armigera* antennae by RT-PCR and RACE techniques based on previous transcriptomic information [38]. Each gene has a corresponding homolog in *H. virescens* and the protein sequences encoded by these genes possess putative seven-transmembrane domains, the typical characteristic of insect odor receptors. Not surprisingly, all these sex pheromone receptor genes showed male-biased expression patterns, since long trichodea sensilla, tuned to sex pheromone components emitted from conspecific females, are significantly abundant in male antennae. Notably, OR13 from *H. virescens and H. armigera* that shared identity up to 91% and specifically tuned to the major sex pheromone component (Z11-16:Ald), were detected as the most abundant transcript in male antennae of both species [6], [21], [49]. The function of OR6 was distinct between species even though they were 88% identical. HvOR6 is narrowly tuned to the second sex pheromone component, Z9-14:Ald, emitted from *H.virescens* female. And HarmOR6 is equally tuned to both Z9-16:Ald and Z9-14:Ald, which recently has been thought to be minor sex pheromone components in *H.armigera*. The function of OR16 has slightly diverged in the two species. Both OR16 genes are sensitively tuned to Z11-16:OH, an attraction inhibitor when added to attraction blends [27], [29]. HarmOR16 also significantly responded to Z9-14:Ald, which may explain why Z9-14:Ald acted as an attraction inhibitor at high concentrations. As in *H.virescens*, no ligand for OR11 and OR15 was identified. The neuron which houses OR11 or OR15 in *H.virescens* didn't respond to tested sex pheromone components in previous electrophysiological recording [22]. The possible explanation is that those genes lost their function during evolution or their ligands aren't included in tested odor panel. OR14 from *H.virescens* has reasonable but weak response to Z11-16:Ac, however, the oocyte that coexpressed HarmOR14/HarmOrco didn't respond to any odorants tested in this study. Our studies didn't involve PBPs, further testing of OR function with PBPs is certainly needed. To date, although no solid experiment has shown that any PBP, with the exception of lush in Drosophila [50], are indispensable for corresponding pheromone receptor's function either in vitro or in vivo, several publications indicated that PBPs could remarkably increase the specificity or sensitivity for some pheromone receptors [6], [51], [52].

In *H.virescens*, in situ hybridization studies for all pheromone receptor genes except for HvOR6 have been carried out and single sensillum electrophysiological recordings were available. Thus, these pheromone receptor genes could be specifically assigned to six neurons of three types of long trichodea sensilla. Further studies in *H.armigera* on colocalization of pheromone receptor genes and electrophysiological function of pheromone-sensitive receptor neurons will make integrated comparison of sex pheromone perception in the two closely related species possible and also significantly improve the importance of this study.

## Supporting Information

Material S1 Primers for gene clone and qRT-PCR expression analyses of HarmPRs.
(XLSX)
